# Idiopathic pulmonary haemosiderosis in paediatric patients: how to make an early diagnosis

**DOI:** 10.1186/s13052-016-0296-x

**Published:** 2016-09-20

**Authors:** Luca Castellazzi, Maria Francesca Patria, Gemma Frati, Andrea Alessandro Esposito, Susanna Esposito

**Affiliations:** 1 Pediatric Highly Intensive Care Unit, Department of Pathophysiology and Transplantation, Università degli Studi di Milano, Fondazione IRCCS Ca’ Granda Ospedale Maggiore Policlinico, Via Commenda 9, 20122 Milan, Italy; 2 Radiology Unit, Fondazione IRCCS Ca’ Granda Ospedale Maggiore Policlinico, Milan, Italy

**Keywords:** Haemoptysis, Idiopathic pulmonary haemosiderosis, Lower respiratory tract infections, Severe anaemia

## Abstract

**Background:**

Idiopathic pulmonary haemosiderosis (IPH) is a rare but potentially lethal condition in paediatric patients. This condition is considered an immune-mediated disorder, but its pathogenesis is still unknown. Idiopathic pulmonary haemosiderosis is characterized by the classical triad of haemoptysis, iron-deficiency anaemia, and diffuse parenchymal consolidation on chest radiology. Unfortunately, this triad of signs is not frequent in children at the onset of this disease, resulting in a delay in diagnosis and a negative outcome.

**Case presentation:**

This case report describes a 4-year-old girl who was admitted for an acute episode of lower respiratory tract infection associated with severe dyspnoea, polypnoea, and severe anaemia (haemoglobin levels, 5.9 g/dL). She had a history of previous similar episodes, with anaemia treated unsuccessfully with iron supplementation and managed through repeated blood transfusions in the acute phase. She did not experience haemoptysis. A computed tomography (CT) scan of the thorax showed ground-glass opacity suggestive of pulmonary haemorrhage. After other causes of intra-alveolar haemorrhage were excluded, IPH was confirmed by the presence of siderophages in bronchoalveolar lavage. Immunosuppressive corticosteroid treatment was immediately started with a good clinical response.

**Conclusion:**

This case highlights the fact that IPH should be suspected in children with recurrent lower respiratory tract infections who have a history of iron-deficiency anaemia who shows no signs of improvement with iron supplementation and may require repeated blood transfusions. The absence of haemoptysis does not exclude the diagnosis of IPH in children. An early and prompt diagnosis is recommended in order to start adequate immunosuppressive treatment.

## Background

Idiopathic pulmonary haemosiderosis (IPH) is a rare clinical condition in paediatric patients. Its incidence is approximately 0.24-1.23 cases per million, with a mortality rate of 50 % [1]. This condition is characterized by recurrent episodes of diffuse alveolar haemorrhage and subsequent abnormal haemosiderin accumulation in pulmonary macrophages, which leads to a thickening of the alveolar basement membrane and, finally, to interstitial fibrosis [2]. Its classical triad of signs at presentation include haemoptysis, iron-deficiency anaemia, and diffuse parenchymal lung consolidation. A delay in diagnosis could lead to a negative prognosis due to the worsening of pulmonary fibrosis and subsequent lethal restrictive lung disease.

In Italy, few cases of IPH have been reported and successful management with immunosuppressive regimens including high dose corticosteroids [2, 3], corticosteroids plus azathioprine [4], or hydroxychloroquine [5] has been described. In addition, a recent review highlighted how the advances in our understanding of IPH have facilitated its diagnosis and management [6].

This case report describes a child with incomplete signs of IPH and discusses presentations that could permit the early diagnosis and management of IPH in paediatric patients.

## Case presentation

A 4-year-old girl was admitted to our paediatric department from another general hospital for an acute episode of lower respiratory tract infection associated with severe dyspnoea, polypnoea, and severe anaemia (haemoglobin level, 5.9 g/dL).

The child was born prematurely at 28 weeks of gestation with an extremely low birth weight (700 g). She had suffered from iron-deficiency anaemia since the age of 3 months and was treated with the oral administration of iron. She also experienced recurrent lower respiratory tract infections, with a significant worsening of anaemia concomitant with four of these episodes (for which she required hospital admission for blood transfusion).

At admission in our ward, the physical findings were as follows: body weight - 10.9 kg; height - 95 cm (<3^rd^ percentile); heart rate - 124 beats/min; respiratory rate - 50 breaths/min; oxygen saturation in the room air - 78 %; blood pressure - 111/71 mmHg; body temperature - 36.6 °C. She was dyspnoeic. Furthermore, blood exams revealed the above-mentioned severe anaemia, a mean corpuscular volume of 67 fL, a reticulocyte count of 3 %, and serum iron (20 μg/dL) and ferritin (8 ng/mL) levels below the normal range. Direct and indirect Coombs tests were negative, and main causes of blood loss and haemolysis were excluded. The coagulation tests and platelet count were normal, and C-reactive protein was mildly increased (3.07 mg/dL; normal value, < 0.5 mg/dL). Serologic tests for immunologic evaluation and autoimmune diseases (i.e., immunoglobulins, anti-nuclear antibodies, anti-DNA antibodies, anti-neutrophil cytoplasmic antibodies, anti-cardiolipin antibodies, anti-smooth muscle antibodies, extractable nuclear antigen, anti-mitochondrial antibodies, anti-myeloperoxidase antibodies, and anti-proteinase 3 antibodies) were performed, and the results were negative. Celiac screening and specific cow’s milk IgE test results were also negative. Bone marrow aspirate showed hyperplastic erythropoiesis. A prompt blood transfusion was performed, oxygen with high-flow nasal cannulae was immediately administered, and i.v. cefotaxime treatment (100 mg/kg/day in three doses for 8 days) was started.

**Fig. 1:**
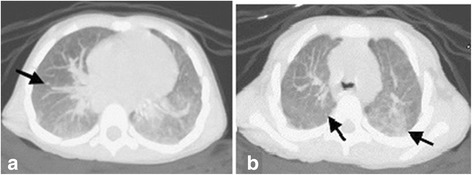
Pulmonary haemorrhage associated with idiopathic pulmonary haemosiderosis. **a** Axial computed tomography (CT) scan at the level of the lower lobes of the thorax (pulmonary window). **b** Axial CT scan at the level of the upper lobes of the thorax (pulmonary window). The images show diffuse ground-glass opacity associated with fine reticulation, findings characteristic of pulmonary haemorrhage. The predominant findings in the acute phase of idiopathic pulmonary haemosiderosis also include diffuse nodules (*black arrows*)

A computed tomography (CT) scan of the thorax was performed and showed a diffuse ground-glass opacity suggestive of pulmonary haemorrhage (Fig. 1). Clinical history (i.e., severe persistent anaemia that worsened during lower respiratory tract infections) and radiologic findings resulted in the suspicion of pulmonary haemosiderosis. A bronchoalveolar lavage was requested and showed the presence of haemosiderin-laden macrophages (siderophages). Thus, idiopathic pulmonary haemosiderosis was diagnosed, and intravenous methylprednisolone (20 mg/kg/day for 3 days) was immediately administered, with a prompt improvement in respiratory symptoms and stabilization of haematological data. Then, oral prednisone (1 mg/kg/day) was started and was well tolerated by the child.

After 6 months of follow-up, the child was still receiving oral prednisone (1 mg/kg/day), had not experienced any exacerbations of the disease and had a haemoglobin level in the normal range.

## Discussion

IPH is a rare clinical condition in paediatric patients. The age at presentation has a bimodal distribution, with a first peak in children less than 5 years old and a second peak in children older than 11 years [1]. No difference in gender distribution is observed in children, whereas this condition mainly occurs in males in the adult population [7]. The rarity of this disease and the variable clinical course results in many diagnostic pitfalls especially in children [6]. The aetiology of IPH is not yet known, although it is considered an immune-mediated disorder. Different triggers in predisposed children are associated with the development of IPH, such as infections (especially respiratory tract infections), cow’s milk protein allergy and celiac disease [8–11]. Some reports have highlighted the frequent association between Down syndrome and the development of IPH probably because children with this condition are more prone to develop autoimmune diseases, especially celiac disease [12]. Recently, IPH was also described in a child with selective immunoglobulin A deficiency, a condition that is known to predispose individuals to the development of autoimmune disorders [13].

IPH is characterized by recurrent episodes of intra-alveolar haemorrhage, which causes the abnormal accumulation of haemosiderin in alveolar macrophages. These macrophages release pro-inflammatory cytokines that cause chronic inflammation and interstitial fibrosis of the lungs [14]. The classical triad of clinical presentation, characterized by iron-deficiency anaemia, recurrent haemoptysis and diffuse parenchymal consolidation on chest radiograph, is not always present in children with IPH [15]. A French study reported that the most frequent clinical features in paediatric patients at disease diagnosis were anaemia and dyspnoea (64 % and 68 %, respectively), whereas haemoptysis occurred in only 50 % of the patients [12]. Haemoptysis is rare in children because in most cases they are not able to expectorate [12, 16]. Iron-deficiency anaemia could precede other signs and symptoms by several months in children with IPH and could be severe enough to necessitate a blood transfusion despite adequate iron supplementation [1, 15, 17, 18]. Other frequent signs in IPH are recurrent lower respiratory tract infections and recurrent tachypnoea [14, 16]. In children who have recurrent lower respiratory tract infections with persistent severe anaemia despite adequate iron supplementation, IPH may be suspected after the exclusion of possible causes of blood loss [1, 14].

A prompt diagnosis of IPH is crucial for early treatment in order to improve the prognosis. Unfortunately, delayed diagnosis is frequently reported, with a delay period of 4 months – 10 years [19]. This delay may be attributed to different reasons, primarily including the insidious onset and a lack of awareness of IPH. The diagnosis of IPH is mainly a diagnosis of exclusion. Diagnosis of IPH is based on exclusion of other causes of intrapulmonary haemorrhage and systemic diseases. When children present with unexplained anemia and bilateral lung infiltrations, pulmonary haemorrhage should be suspected [6]. In addition, autoimmune diseases and coagulation disorders should be excluded [20]. Furthermore, considering the association of IPH with celiac disease and cow’s milk protein allergy, screening for these conditions should be performed in all patients [12]. The gold standard for the diagnosis of IPH is considered lung biopsy, but this method is invasive and not practiced everywhere in children [6]. Even our patient did not perform lung biopsy, although this diagnostic method is useful to exclude other causes of interstitial lung diseases. Another specific and sensitive method for the diagnosis of IPH is bronchoalveolar lavage analysis, which permits the confirmation of the diagnosis with a high percentage of haemosiderin-laden macrophages (siderophages) [21]. Recently, some authors highlighted the fact that the search for siderophages can also be completed by gastric lavage fluid analysis, which is a simple and reliable test that can be used in children [1].

Immunosuppressive agents are the treatment of choice for IPH, despite the lack of large controlled studies on this topic. High doses of steroids are considered the first line of treatment in IPH to reduce episodes of haemorrhage and bleeding. In cases that are not completely controlled by steroids or in those patients who experience side effects to these drugs, other immunosuppressive agents such as azathioprine, chloroquine, cyclophosphamide and 6-mercaptopurine have to be considered [12, 22–25]. The prognosis of patients with IPH, which has improved in the last years, is influenced by several factors, including the time of diagnosis, the early initiation of treatment and the presence of comorbidities [15].

## Conclusion

In conclusion, this case report highlights the fact that IPH should be suspected in children with recurrent lower respiratory tract infections who have a history of iron-deficiency anaemia, who shows no signs of improvement with iron supplementation and may require repeated blood transfusions. Haemoptysis, which is considered a classical sign of IPH, is rare in children, and its absence does not exclude the diagnosis of IPH. An early and prompt diagnosis is recommended to start adequate immunosuppressive treatment.

## Abbreviations

CT: Computed tomography
IPH: Idiopathic pulmonary haemosiderosis
